# HIV Cure Strategies: How Good Must They Be to Improve on Current Antiretroviral Therapy?

**DOI:** 10.1371/journal.pone.0113031

**Published:** 2014-11-14

**Authors:** Paul E. Sax, Alexis Sypek, Bethany K. Berkowitz, Bethany L. Morris, Elena Losina, A. David Paltiel, Kathleen A. Kelly, George R. Seage, Rochelle P. Walensky, Milton C. Weinstein, Joseph Eron, Kenneth A. Freedberg

**Affiliations:** 1 Division of Infectious Diseases, Brigham and Women’s Hospital, Boston, Massachusetts, United States of America; 2 Department of Orthopedic Surgery, Brigham and Women’s Hospital, Boston, Massachusetts, United States of America; 3 Harvard University Center for AIDS Research, Harvard University, Boston, Massachusetts, United States of America; 4 Harvard School of Public Health, Harvard University, Boston, Massachusetts, United States of America; 5 Division of General Medicine, Department of Medicine, Massachusetts General Hospital, Boston, Massachusetts, United States of America; 6 Division of Infectious Diseases, Department of Medicine, Massachusetts General Hospital, Boston, Massachusetts, United States of America; 7 Medical Practice Evaluation Center, Department of Medicine, Massachusetts General Hospital, Boston, Massachusetts, United States of America; 8 Department of Biostatistics, Boston University School of Public Health, Boston, Massachusetts, United States of America; 9 Department of Epidemiology, Boston University School of Public Health, Boston, Massachusetts, United States of America; 10 Yale School of Public Health, New Haven, Connecticut, United States of America; 11 Division of Infectious Disease, School of Medicine, University of North Carolina at Chapel Hill, Chapel Hill, North Carolina, United States of America; University of Pittsburgh, United States of America

## Abstract

**Background:**

We examined efficacy, toxicity, relapse, cost, and quality-of-life thresholds of hypothetical HIV cure interventions that would make them cost-effective compared to life-long antiretroviral therapy (ART).

**Methods:**

We used a computer simulation model to assess three HIV cure strategies: Gene Therapy, Chemotherapy, and Stem Cell Transplantation (SCT), each compared to ART. Efficacy and cost parameters were varied widely in sensitivity analysis. Outcomes included quality-adjusted life expectancy, lifetime cost, and cost-effectiveness in dollars/quality-adjusted life year ($/QALY) gained. Strategies were deemed cost-effective with incremental cost-effectiveness ratios <$100,000/QALY.

**Results:**

For patients on ART, discounted quality-adjusted life expectancy was 16.4 years and lifetime costs were $591,400. Gene Therapy was cost-effective with efficacy of 10%, relapse rate 0.5%/month, and cost $54,000. Chemotherapy was cost-effective with efficacy of 88%, relapse rate 0.5%/month, and cost $12,400/month for 24 months. At $150,000/procedure, SCT was cost-effective with efficacy of 79% and relapse rate 0.5%/month. Moderate efficacy increases and cost reductions made Gene Therapy cost-saving, but substantial efficacy/cost changes were needed to make Chemotherapy or SCT cost-saving.

**Conclusions:**

Depending on efficacy, relapse rate, and cost, cure strategies could be cost-effective compared to current ART and potentially cost-saving. These results may help provide performance targets for developing cure strategies for HIV.

## Introduction

Combination antiretroviral therapy (ART) durably controls HIV replication and halts progression of clinical HIV disease in the vast majority of patients who receive and continue treatment [1]. Projected survival for people with HIV is now estimated to be several decades. Some reports suggest that survival for people with HIV on successful therapy approaches that of those without infection if therapy is initiated early and HIV suppression is sustained [2].

Despite the remarkable success of treatment, ART nonetheless has many limitations. Although much less toxic than earlier regimens, current treatment still may be associated with cardiovascular, renal, bone, and other complications [3], [4]. The inflammation and immune activation that persist in many patients on suppressive ART may have long-term negative consequences [5]. Therapy in the US and Europe remains costly, and, because not curative, it must be continued indefinitely [6], [7]. Successful ART also does not eliminate the stigma associated with HIV infection [8].

The first report of successful HIV cure after allogeneic stem cell transplant for acute leukemia demonstrated that eradicating HIV from an individual is viable [9]. While allogeneic transplant in the absence of usual indications carries substantial risk, cost, and post-transplant consequences of chronic immunosuppression, other strategies are being studied that could potentially cure HIV and be practically deployed [10]–[12]. In this analysis we aim to establish thresholds of efficacy, toxicity, durability, cost, and quality of life necessary for a cure strategy to compare favorably with current antiretroviral therapy in the United States.

## Methods

### Analytic Overview

To analyze the potential life expectancy and cost-effectiveness of HIV cure strategies under study, we utilized the Cost-Effectiveness of Preventing AIDS Complications (CEPAC) model, a Monte-Carlo microsimulation of HIV disease and treatment [13]. We completed a ‘what if’ analysis, in order to understand the possible role of HIV cure strategies as they are developed. Model outputs included life expectancy, quality-adjusted life expectancy, and lifetime costs (2012 USD), all discounted to present value at 3% annually [14]. Incremental cost-effectiveness ratios (ICERs) were calculated by comparing each hypothetical cure strategy to the standard of care, lifelong ART. We determined parameter thresholds at which potential cure strategies were either cost-effective, defined as ICERs <$100,000/quality-adjusted life year (QALY), or cost-saving compared to current ART [15].

### Strategies Evaluated

We evaluated three hypothetical HIV cure strategies: a “low efficacy,” “low risk” gene therapy approach (Gene Therapy); a “moderate efficacy,” “moderate risk” chemotherapy approach (Chemotherapy); and a “high efficacy,” “high risk” allogeneic stem cell transplant (SCT). Costs of these strategies would likely vary widely and are currently uncertain.

The Gene Therapy strategy was modeled after the use of zinc finger nucleases to modify the CCR5 receptor on the surface of CD4 cells [12]. Patients undergo pheresis, their cells are modified using zinc finger nucleases, and re-infused with the goal of establishing a CCR5-negative cell population that is resistant to HIV infection. Based on preliminary reports, this type of procedure would have lower risk and toxicity than Chemotherapy and SCT and, we assumed, lower likelihood of achieving cure [16]–[19]. Simulated patients were modeled to receive the benefit of cure one month after Gene Therapy, if effective. Input parameters for all strategies were varied widely in sensitivity analysis, as described below.

The Chemotherapy intervention was derived from both in vitro and in vivo experiments using histone deacetylase inhibitors (such as vorinostat) to stimulate and eliminate the HIV viral reservoir [10]. Simulated patients received ART combined with Chemotherapy for 96 weeks, after which, if effective, they had the benefit of cure. There was increased cost and toxicity for the chemotherapy-based administration of vorinostat [17], [20].

SCT had the highest assumed risk of mortality and toxicity, but was assumed the most effective. Simulated SCT patients received the benefit of cure in the first month after successful transplant.

### The Cost-Effectiveness of Preventing AIDS Complications (CEPAC) Model

Simulations were performed using the CEPAC model, a widely-published, validated state-transition microsimulation of HIV disease [13]. HIV natural history is modeled as a series of monthly transitions between health states characterized by CD4 count and HIV RNA. Without treatment, patients’ CD4 counts decline according to a viral load-dependent trajectory [21]. Patients are also subject to age- and sex-specific non-HIV-related mortality [22].

Once patients initiate ART, the probability of virologic suppression and subsequent CD4 count increases, with the greatest CD4 gain occurring in the first two months [23]. CD4 count gains are associated with reduced risk of developing opportunistic infections and HIV-related death. Patients’ HIV RNA and CD4 counts are routinely monitored to detect treatment failure. Upon virologic rebound, patients switch to the next available ART regimen. Costs of HIV treatment and care are from the health system perspective and derived from HIV Research Network data and the Medicare fee schedule [24]–[27].

### Cure Simulation

This analysis focused on patients who had received fully suppressive first-line ART for one year and were thereby eligible for a cure strategy, as is the case in planned or ongoing cure trials [28]. We maintained the CD4 benefit associated with virologic suppression for each cure strategy. With each cure regimen, patients faced strategy-specific probabilities of achieving cure as well as toxicity, quality of life (QOL) decrements and increases (associated with both toxicity and the regimen itself), and monthly probabilities of relapse. Additionally, patients accrued strategy-specific intervention costs. Cured patients were no longer subject to monthly probabilities of opportunistic infections and AIDS-related death, but were subject to monthly probabilities of relapse and subsequent return to ART. After cure, patients faced monthly probabilities of non-AIDS mortality and accrued monthly costs for routine care and continued HIV RNA monitoring for relapse. Patients who failed cure, or later relapsed after cure, resumed first-line ART, followed by additional ART regimens if virologic failure occurred later.

### Model Inputs and Analysis

We used the CEPAC model itself to determine the distribution of CD4 counts in the eligible population by simulating a cohort of patients entering the model with the age, sex, and CD4 count distribution of HIV-infected patients in North America at care presentation. Patients were given a first-line ART regimen of efavirenz, tenofovir, and emtricitabine for one year [29]. Per current guidelines, all patients received ART, regardless of CD4 count [30]. Following one year on suppressive ART, patients became eligible for a cure intervention, beginning these cure strategies with mean CD4 count of 564/µl (SD 250/µl), based on this initialization.

Patients assigned to a cure intervention were subject to a strategy-specific probability of being cured (Table 1). All efficacies were hypothetical, since cure interventions do not currently exist. Cured patients had undetectable viremia for the duration of their lifetimes, unless they relapsed. We assumed relapse rates were highest during the first five years after a cure intervention (0.5%/month); after five years the relapse rate was reduced by one half (0.25%/month). Relapse was detected through routine virologic monitoring. Both acute and chronic non-fatal toxicities resulted in a QOL decrement of 0.04, which lasted one month for acute non-fatal toxicities and until the patient failed the cure strategy for chronic toxicities [31]. Because the cohort was comprised only of patients virologically suppressed on first-line ART for one year, we assumed high rates of virologic re-suppression after a failed cure intervention. Those patients were also at risk for later virologic failure, at a rate of 0.13%/month [32]. Costs associated with each of the interventions and their associated toxicities were based on reported costs for similar procedures for other conditions (Table 1). In the base case, we assumed no additional QOL benefit related to achieving HIV cure compared to being on effective ART. In sensitivity analyses, we considered scenarios in which cured patients had an increase in their QOL from the base case. Any QOL benefit was suspended if the patient relapsed and re-initiated ART.

**Table 1:** Parameter inputs for a model-based analysis of potential HIV cure strategies.

Variable: Base Case(Range)	GeneTherapy	Chemotherapy	Stem CellTransplant	References
**Cohort Characteristics**				
CD4 count, mean cells/µl (SD)	564 (250)	564 (250)	564 (250)	See Methods^a^
Age, mean years (SD)	44 (12)	44 (12)	44 (12)	[29]
Percent male	84	84	84	[29]
**Cure Characteristics**				
Efficacy (%)	10.0(10.0–90.0)	20.0(10.0–90.0)	70.0(10.0–90.0)	Assumptions
Monthly relapse rate (%),early/late	0.50/0.25(0.0–2.0)	0.50/0.25(0.0–2.0)	0.50/0.25(0.0–2.0)	Assumptions
Initial cost ($)	100,000(50,000–200,000)	12,400/month^b^(6,200–24,800)	150,000(75,000–300,000)	Assumptions based on[20], [33], [34], [36]/[20]/[36]
Additional cost ($, while on cureregimen only)	2,000/month^c^	2,000/month^c^	1,000/month^d^(for 6 months)	[20], [34]/[20], [34]/[37]
Fatal Toxicity				
Probability (%)	0.0	1.2	5.0	Assumption based on [16]/[17]/[35]
Cost ($)	–	63,110	63,110	Derived from [24], [25], [27], [46]
Acute Non-fatal Toxicity				
Probability (%)	25.0	6.0	47.3	Assumption based on Ivacaftor packageinsert [16]/[18]/[19]
Cost ($)	50	3,100	18,700	[25]/[47]/Derived from [48]
Chronic Non-fatal Toxicity				
Probability (%)	0.0	5.8	37.2	Assumption based on [16]/[18]}/[19]
Cost ($)	–	1,040	1,900	[49]/Derived from [50]

**SD:** standard deviation; **QOL:** quality-of-life.

a Determined through initialization run of simulated cohort; ^b^For 24 months based on vorinostat; ^c^For monthly antiretroviral therapy, derived from weighted averages of current therapies until gene- or chemo-therapy is complete; ^d^For immunosuppressive agents, including methotrexate with tacrolimus.

Gene Therapy was assumed to have an efficacy of 10.0% with no risk of fatal toxicity [16]. Patients incurred a 25.0% risk of acute, non-fatal toxicity (e.g., headache or oropharyngeal pain) lasting for one month [16]. While receiving Gene Therapy, patients incurred an immediate cost of $100,000, based on current estimates for gene therapies, plus $2,000 for continued ART (from weighted average of current drug prices) during the month they received Gene Therapy [20], [33], [34]. This intervention cost was based on ivacaftor, an oral cystic fibrosis medication that acts on the genetic mutation causing the disease [20].

Chemotherapy was assumed to have an efficacy of 20.0%, and 1.2% probability of fatal toxicity [17]. Patients incurred a 6.0% risk of acute non-fatal toxicity and 5.8% risk of chronic non-fatal toxicity [17], [18]. Chemotherapy was modeled as a 96-week course (24 months) with monthly costs of $12,400; $2,000/month was included for maintenance ART [17]. At any point in the 96-weeks patients could fail ART and experience HIV virologic rebound. Patients who had not experienced ART failure during the 96 weeks could be cured at the end of that period (assumed efficacy 20.0%).

SCT was assumed to have an efficacy of 70.0%, with 5.0% mortality from the procedure [35]. Patients had a 47.3% probability of acute graft-versus-host disease and 37.2% probability of chronic graft-versus-host-disease [19]. The initial cost of the transplant was assumed to be $150,000 with monthly costs of $1,000 for six months for immunosuppressive medications [36], [37].

### Sensitivity Analysis

Because the focus of this analysis was on strategies under research and development, we conducted extensive sensitivity analysis on all cure parameters to identify those most important in changing the main conclusions. For each cure strategy and parameter, we determined thresholds at which the strategy would become cost-effective at a threshold of $100,000/QALY, as well as become cost-saving compared to ART. For sensitivity analyses involving relapse rates, early (≤5 years) and late (>5 years) relapse rates were varied together. Recognizing the impact a cure might have on patients’ well-being (physical, emotional, and social), we also conducted sensitivity analysis on health-related QOL, both prior to and following HIV cure. Due to the major toxicity, including fatal toxicity, involved in SCT, we focused the QOL sensitivity analysis on the Gene Therapy and Chemotherapy strategies.

### Ethics Statement

This study was reviewed and approved by the Partners Heath Care Human Research Committee (Protocol 2000P001927), Boston, Massachusetts, USA, as it was determined to meet the criteria for exemption from human studies. A waiver for written informed consent from participants was not necessary because only secondary data were used in this study and no human subjects were involved. Secondary patient data that serve as our model inputs were anonymized and de-identified prior to analysis.

## Results

### Base Case Scenarios

The standard of care (lifelong ART) had a discounted projected life expectancy of 19.0 years (16.4 QALYs) and discounted lifetime cost of $591,400. Undiscounted life expectancy with standard of care was 32.3 years, compared to 32.8, 32.3, and 32.6 years, for Gene Therapy, Chemotherapy, and SCT under the base case set of assumptions. Gene Therapy (10% efficacy) resulted in a discounted life expectancy of 19.3 years (16.6 QALYs) and increased discounted lifetime costs to $658,700, for an ICER of $330,600/QALY gained compared to continued ART. Chemotherapy (20% efficacy) led to a discounted life expectancy of 19.0 years (16.4 QALYs) and discounted lifetime cost of $807,300, and was more expensive and less effective than ART. SCT resulted in a discounted life expectancy of 19.0 years (16.3 QALYs) and increased costs to $607,400; it was also more expensive and less effective than ART (Table 2).

**Table 2:** Base case results of an analysis of hypothetical HIV cure strategies*.

Strategy	Discounted LifeYears(Undiscounted)	DiscountedQALYs	Cost ($)	Incremental Cost-effectiveness comparedto standard of care ($/QALY)
Standard of care ART	19.0 (32.3)	16.4	591,400	–
Gene Therapy	19.3 (32.8)	16.6	658,700	330,600
Chemotherapy	19.0 (32.3)	16.4	807,300	Dominated
Stem Cell Transplant	19.0 (32.6)	16.3	607,400	Dominated

*Based on assumptions for efficacy, durability, toxicity, and cost in Methods and Table 1. Life expectancy, QALYs, and costs all discounted at 3%/year. **ART:** antiretroviral therapy; **QALY:** Quality-adjusted life year; **Dominated:** Less effective and more costly than the standard of care ART strategy.

### One-way Sensitivity Analyses

With efficacy increased to 22% and other inputs remaining the same, Gene Therapy had an ICER <$100,000/QALY, and at an efficacy of 34% became cost-saving, relative to ART (Table 3). With a reduced cost of $54,000, Gene Therapy achieved an ICER<$100,000/QALY gained even at 10% efficacy; it was cost-saving at $34,000. Chemotherapy was not cost-effective unless efficacy increased to 88% and was not cost-saving at any efficacy. Varying any other single parameter within reasonable limits did not result in Chemotherapy reaching thresholds for cost-effectiveness or cost savings (Table 3). The efficacy threshold for SCT was 79% to achieve cost-effectiveness and 80% to achieve cost savings. Reducing fatal toxicity to 3.0% from 5.0% also led to SCT becoming cost-effective (Table 3).

**Table 3:** Threshold which key parameters would need to reach for each type of HIV cure strategy to be cost-effective (ICER<$100,000/QALY gained) or cost-saving.

Parameter	Base case value	ICER<$100,000/QALY gained	Cost-saving
**Gene Therapy** (base case ICER: $330,600/QALY gained)			
Efficacy (%)	10	22	34
Fatal Toxicity (%)	0.0	None	None
Monthly relapse rate (%),early (late)	0.5/0.25	None	None
Intervention cost ($)	100,000, one-time	54,000, one-time	34,000, one-time
**Chemotherapy** (base case ICER: Dominated)			
Efficacy (%)	20	88	None
Fatal Toxicity (%)	1.2	None	None
Monthly relapse rate (%),early (late)	0.5/0.25	None	None
Intervention cost ($)	12,400/month,for 24 months	*	*
**Stem Cell Transplant** (base case ICER: Dominated)			
Efficacy (%)	70	79	80
Fatal Toxicity (%)	5.0	3.0	None
Monthly relapse rate (%),early (late)	0.5/0.25	None	0.25/0.125
Intervention cost ($)	150,000, one-time	*	*

**ICER:** incremental cost-effectiveness ratio; **QALY:** quality-adjusted life year; **QOL:** quality of life; **Dominated:** strategy was less effective and more expensive than current ART.

*Cost reductions led to the strategy being less effective and less expensive than current ART. One could calculate an ICER for ART compared to Chemotherapy or Stem Cell Transplant, but it is not clinically plausible that these strategies would be used if they resulted in worse outcomes than standard of care with ART, even if they saved money by avoiding the costs of lifelong ART.

### Multiway Sensitivity Analyses

**Figure 1:**
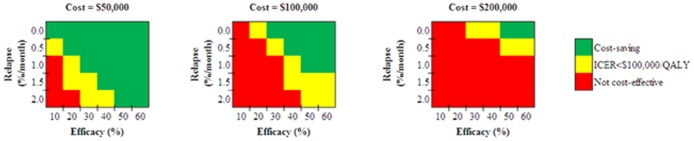
Gene Therapy compared to standard of care ART. The figure depicts the cost-effectiveness of Gene Therapy compared to standard of care ART as a function of the three influential parameters identified via the one-way sensitivity analysis in Table 3: cost, relapse rate, and efficacy. In each panel, the horizontal axis denotes efficacy while the vertical axis denotes the relapse rate. Inside each panel, the shading denotes the resultant cost-effectiveness finding, ranging from cost-saving (green), through cost-effective (with an ICER<$100,000/QALY, yellow), to not cost-effective (≥$100,000/QALY or more expensive and less effective than ART, red). **ART:** antiretroviral therapy; **ICER:** incremental cost-effectiveness ration; **QALY:** quality-adjusted life year.

With no relapse risk, Gene Therapy was cost-saving with efficacy of at least 30%. With increasing relapse rates, higher efficacy was required to achieve cost savings. At a decreased cost of $50,000, Gene Therapy became cost-effective at the base case values for relapse and efficacy and cost-saving with lower relapse rates or higher efficacies (Figure 1). At increased cost of $200,000, the intervention was not cost-effective compared to standard of care ART for almost all combinations of input parameters (Figure 1).

**Figure 2:**
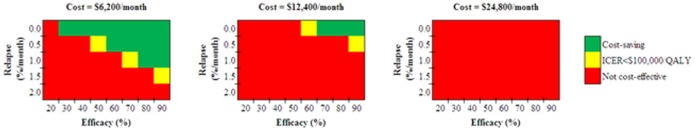
Chemotherapy compared to standard of care ART. The figure depicts the cost-effectiveness of Chemotherapy compared to standard of care ART as a function of the three influential parameters identified via the one-way sensitivity analysis in Table 3: cost, relapse rate, and efficacy. In each panel, the horizontal axis denotes efficacy while the vertical axis denotes the relapse rate. Inside each panel, the shading denotes the resultant cost-effectiveness finding, ranging from cost-saving (green), through cost-effective (with an ICER<$100,000/QALY, yellow), to not cost-effective (≥$100,000/QALY or more expensive and less effective than ART, red). **ART:** antiretroviral therapy; **ICER:** incremental cost-effectiveness ration; **QALY:** quality-adjusted life year.

For Chemotherapy, at the base case cost and relapse rate of greater than 0.5%/month, the intervention was never cost-effective (Figure 2). With no relapse risk, the intervention was not cost-effective at efficacies of 20–50% but was cost-saving at efficacies above 60%. If the cost was halved ($6,200/month), Chemotherapy was cost-saving at substantially lower efficacies and higher relapse rates than in the base case. For example, at this decreased cost, Chemotherapy was cost-saving with relapse rate of 0.5%/month with efficacy 60%. If the cost of Chemotherapy was doubled to $24,800/month, it was not cost-effective with any combination of efficacy (20–90%) and relapse rate (0.0–2.0%). The window for cost-effectiveness was narrow; with most parameter combinations, Chemotherapy was either cost-saving or not cost-effective.

**Figure 3:**
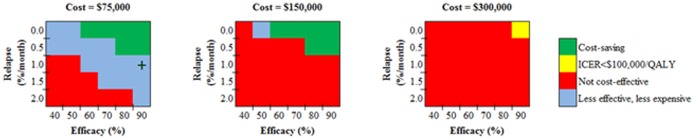
Stem Cell Transplantation compared to standard of care ART. The figure depicts the cost-effectiveness of Stem Cell Transplantation compared to standard of care ART as a function of the three influential parameters identified via the one-way sensitivity analysis in Table 3: cost, relapse rate, and efficacy. In each panel, the horizontal axis denotes efficacy while the vertical axis denotes the relapse rate. Inside each panel, the shading denotes the resultant cost-effectiveness finding, ranging from cost-saving (green), through cost-effective (with an ICER<$100,000/QALY, yellow), to not cost-effective (≥$100,000/QALY or more expensive and less effective than ART, red). Instances where the intervention is both less expensive and less effective than ART are denoted in blue, but most were not cost-effective because the ICER of ART was <$100,000/QALY compared to SCT. The plus sign indicates a strategy that had an ICER for ART compared to SCT >$100,000/QALY gained. **ART:** antiretroviral therapy; **ICER:** incremental cost-effectiveness ration; **QALY:** quality-adjusted life year.

In most sensitivity analyses, SCT was not cost-effective. In selected cases where the cost was extremely low or efficacy very high, SCT became cost-saving (Figure 3). For one parameter combination, SCT was less effective and less expensive than ART, but it was not cost-effective because the ICER of ART was <$100,000/QALY compared to SCT. If the cost of SCT was halved ($75,000), the combinations where the intervention was cost-saving remained roughly the same, but several scenarios that were not cost-effective in the base case became less expensive and less effective than ART.

With an efficacy of 10% for Gene Therapy, improving QOL to a utility of 1.00 (i.e., the equivalent of perfect health) after successful cure would be insufficient to achieve an ICER <$100,000/QALY gained. With efficacy of 20%, however, an ICER <$100,000/QALY gained could be achieved if patient utility following cure increased from 0.85 to 0.88, or the equivalent of facing a 3% decreased risk of death every year. For efficacies of 30% or more, the Gene Therapy strategy would always be cost-effective, regardless of whether the cure had any impact on QOL. At the base-case QOL utility of 0.85, Chemotherapy was not cost-effective at any efficacy below 60%, even with the maximum QOL improvement. At an efficacy of 60% for Chemotherapy, cost-effectiveness could be achieved if patient utility following cure increased from 0.85 to 0.97. If the baseline QOL utility while living with HIV were 0.50, Chemotherapy would not reach the cost-effectiveness threshold of <$100,000/QALY at cure efficacies below 40%. At cure efficacy of 40%, Chemotherapy would achieve an ICER below $100,000/QALY gained with improvement in QOL utility to 0.88. If we used ICER thresholds below $150,000 or $200,000 per QALY gained to define cost-effectiveness, there were no appreciable changes in results [15].

## Discussion

With intense pre-clinical investigation underway towards finding a cure for HIV, we sought to evaluate the cost-effectiveness of three potential HIV cure approaches, each compared to standard of care ART. We used a variety of assumptions, anchored in published data on gene-targeted therapy, chemotherapy, and stem cell transplant for diseases other than HIV. By doing extensive sensitivity analyses on efficacy, toxicity, relapse rates, and cost, we defined a range of benchmarks that might justify the adoption of a cure strategy, and identified combinations of parameters under which these could potentially be cost-effective or cost-saving. For a Gene Therapy approach, modest increases in efficacy (above 10%) or moderate decreases in cost (below $100,000), led to this strategy being cost-saving compared to ART. For Chemotherapy and SCT, the inventions became cost-saving with very high efficacies and low relapse rates.

We found that changes in efficacy, relapse rates, and/or cost rapidly moved the strategies from being worse than ART to being cost-saving – that is, to being both equally or more effective and less costly. The range in which any strategy would be cost-effective but not cost-saving is narrow (Figures 1–3, yellow area). High initial costs of cure strategies could be justified, and would save money, if (and essentially only if) the strategy eliminates the lifetime cost of ART. For example, with an initial cost of $100,000 and an efficacy of 34%, the Gene Therapy strategy is cost-saving compared to ART, even if all other assumptions remain the same. In such a scenario, identification of conditions that could theoretically increase the likelihood of cure – such as ART started during acute infection, or heterozygosity of the CCR5delta32 gene – would make a cure strategy even more attractive [38]. Alternatively a substantial decrease in the cost of lifelong ART would make these interventions less cost-effective.

It is possible that combination approaches to cure may be needed to improve efficacy [39]. These would, nonetheless, each have some combination of efficacy, toxicity, and cost. The value in terms of cost-effectiveness, compared to ART, can be inferred from those combinations as shown in Figures 1–3. Further, some lower-risk interventions, such as zinc finger nucleases, could also have higher efficacy than other interventions. If so, then they would both be more effective and less costly, and thus ‘dominant’ from a cost-effectiveness perspective, compared to those other interventions, such as HDAC inhibitors.

No published studies to date have examined the cost-effectiveness of hypothetical HIV cure strategies in comparison to ART. Similar model-based analyses have, however, been done for other previously unproven strategies in HIV, including therapeutic and preventive HIV vaccines and pre-exposure prophylaxis (PrEP) [40]–[42]. These analyses have been used to design subsequent vaccine and PrEP research. In the case of PrEP, modeled results before proven efficacy closely matched the outcome of some later trials [43].

At present, strategies to cure HIV have only progressed to the proof of concept stage. Given this early stage, current complexity, anticipated cost, and possible risks, a cure strategy will not be ready for implementation anytime soon. However, this analysis suggests that potential HIV cure strategies must be moderately effective and have low toxicity and low relapse rates to compare favorably to standard of care ART. The optimal cost threshold for such strategies will depend on both the likelihood of durable cure (initial efficacy and subsequent relapse rate) and the cost of ART. As initial efforts at cure are developed, this work can help investigators determine the efficacy and toxicity targets which would make the strategies attractive. Further, if any cure strategies are proven effective, the results of this analysis can help inform policymakers as to their appropriate role. This issue has recently been highlighted by the high efficacy and cost of new HCV cures [44].

From a societal and quality-of-life perspective, with a base case utility of 0.85 for patients doing well on ART, improvements in quality of life after cure do not have a major impact on cost-effectiveness. However, many might argue that there is an important psychological, social, and emotional distinction to be drawn between curing HIV and controlling it via therapy.

Our study has several limitations. The most important is that HIV cure interventions do not yet exist, so model parameters such as efficacy, mortality, cost, and relapse rates were assumed using specific data wherever possible and then varied widely. The effect of cure strategies on the incidence and severity of “non-HIV” complications, such as malignancies, heart disease, and other chronic non-communicable diseases was not included; one might anticipate either an increase or decrease in these complications, based on the strategy employed. If non-AIDS events are driven primarily by HIV-mediated immune activation and inflammation, then curing HIV would presumably ameliorate these processes. In addition, adverse effects of antiretroviral drugs would also be eliminated. By contrast, some of the treatments proposed for HIV cure may themselves increase risks of non-AIDS events. For example, some are analogous to cancer chemotherapy, and such treatments may increase the risk of secondary malignancies; radiation used for stem cell transplant could also raise cardiovascular risk; and alteration in stem cells could also increase the long-term risk of cancers. The demographics of the suppressed patients eligible for cure interventions were based on the demographics of the population presenting to care in the United States and may not be completely representative of those who achieve suppression after one year. Since we modeled only patients virologically suppressed after a year, this represents the most adherent subset of patients. If cure strategies were utilized in a broader group of patients, such as those with early infection, the strategies might be more or less effective and cost-effective compared to ART, depending on the requirements of the particular cure strategy. Gene therapy may require stem cell modification to achieve cure, which could increase the risk of rare but substantial toxicity of cancer induction; this risk was not included. Although we did include relapse rates – indicating a later chance of HIV viral rebound after initial cure – we did not include the possibility of re-infection among cured patients, which has been documented after successful HCV cure [45]. Adding this possibility would make any cure strategy less attractive. Increased use of newer, more effective branded therapies, however, may keep the costs of ART in their current range [20].

In summary, the key determinants of the cost-effectiveness of HIV cure strategies, compared to current antiretroviral therapy, are initial efficacy, toxicity, relapse rate, and cost. Potential cure strategies must have moderate efficacy, low toxicity, and relatively low risk of relapse to be cost-effective and, in combination, would likely be cost-saving.
