# Correction: HIV Cure Strategies: How Good Must They Be to Improve on Current Antiretroviral Therapy?

**DOI:** 10.1371/journal.pone.0117762

**Published:** 2015-01-26

**Authors:** The PLOS ONE Staff

The following information is missing from the Funding section: This study was supported by NIH grant number R56 AI114617 to PES and KAF.
